# Microcirculation

**DOI:** 10.1155/2012/867176

**Published:** 2012-10-04

**Authors:** Michael Piagnerelli, Can Ince, Arnaldo Dubin

**Affiliations:** ^1^Department of Intensive Care, CHU-Charleroi, Université Libre de Bruxelles, 92, Boulevard Janson, 6000 Charleroi, Belgium; ^2^Experimental Medicine Laboratory, CHU-Charleroi, 6110 Montigny-le-Tilleul, Belgium; ^3^Department of Intensive Care, Erasmus Medical Center, University Medical Center, P.O. Box 2040, 3000 CA Rotterdam, The Netherlands; ^4^Department of Translational Physiology, Academic Medical Center, University of Amsterdam, Meibergdreef 9, 1105 AZ Amsterdam, The Netherlands; ^5^Servicio de Terapia Intensiva, Sanatorio Otamendi y Miroli 870, C1115AAB Buenos Aires, Argentina; ^6^Catedra de Farmacologia Aplicada, Facultad de Ciencias Médicas, Universidad Nacional de La Plata, Argentina

The microcirculation is the part of the circulation where oxygen, nutrients, hormones, and waste products are exchanged between circulating blood and parenchymal cells.The microcirculation includes not only all the vessels with a diameter <100 *μ*m but also the interactions between blood components (circulating cells, coagulation factors), the vessels lined by the endothelium, and the glycocalyx.

Over the last decade, especially since the development of new techniques such as orthogonal polarized spectral (OPS) and sidestream dark field (SDF) imaging, we have been able to assess alterations in the microcirculation of critically ill patients at the bedside [1–4]. From the various studies, it is clear that all components of the microcirculation are altered early in critical illness, especially during sepsis. Persistence of these alterations is associated with increased morbidity and a poor outcome [2, 4, 5]. Interestingly, these alterations are not correlated with systemic hemodynamics [6], making monitoring the microcirculation of particular interest for titrating potential therapies. 

So, should we all be assessing the microcirculation at the bedside and use it to guide therapy in all critically ill patients? Unfortunately, we are not yet ready for this step! Indeed, several questions need to be answered before we try to modulate the microcirculation with any therapeutic intervention. In this special issue, several recent studies in this field are published to try and provide some responses to these remaining questions. 

Before microcirculatory monitoring can become widespread, it needs to be standardized: first, in terms of imaging the sublingual microcirculation, and second, in terms of quantifying the alterations observed. N. A. R. Vellinga et al. suggest the development of a picture database from 36 intensive care units (ICUs) worldwide. These authors called their network: microSOAP (Microcirculatory Shock Occurrence in Acutely ill Patients). The aim of this multicenter, observational study was to collect 500 images from critically ill patients and to estimate the prevalence of microcirculatory alterations in ICU patients, related to conventional clinical and hemodynamic variables. Moreover, this database could serve as a source for further investigations. 

Despite a roundtable involving experts in the field [7], scoring of microcirculatory alterations remains controversial [8, 9] and could limit the expansion of this technique. Indeed, if different scoring techniques are used in different studies, it is difficult to compare studies and patients. In this special issue, M. O. Pozo et al. compared different methods of calculating the microvascular flow index (MFI). This index is commonly used to semiquantitatively characterize the velocity of microcirculatory perfusion as absent, intermittent, sluggish, or normal [7, 10]. Three approaches are described to compute the MFI: (1) the average of the predominant flow in each of the four quadrants (MFI by quadrants), (2) direct assessment during bedside video acquisition (MFI point of care), and (3) the mean value of the MFIs determined in each individual vessel (MFI vessel by vessel). In this study, performed by analyzing 100 pictures from septic patients, the best correlations were between the MFI vessel by vessel and RBC velocity (*r* ^2^ : 0.61, *P* < 0.0001) and between the MFI vessel by vessel and the fraction of perfused small vessels (*r* ^2^ : 0.96, *P* < 0.0001). Although MFI measurement reflects the magnitude of microvascular perfusion, the different approaches are not interchangeable. As noted by the authors, however, although the MFI vessel by vessel approach may seem to be preferable, it is time consuming and does not facilitate use of the technique at the bedside. 

Also in this issue, E.–S. Tripodaki et al. and A. P. C. Top et al. introduce new pieces into the puzzle of the relationship of the microcirculation and systemic hemodynamics. First, Tripodaki et al. evaluate the relationship of muscle microcirculation to systemic parameters and outcome after cardiac surgery. The authors studied the microcirculation using near-infrared spectroscopy (NIRS) and the vascular occlusion technique. They observed good correlations between NIRS-derived variables and cardiac output, lactate, and mortality. These relationships have been described previously in septic shock [11] but this is the first time they have been demonstrated in cardiac surgery patients. The dependence of the microcirculation on systemic hemodynamics is a controversial issue. In septic shock, sublingual microvascular perfusion is independent of either cardiac output or blood pressure [4]; therefore, the microcirculation may behave as an independent compartment of the cardiovascular system. Increasing blood pressure with norepinephrine, however, did affect the sublingual microcirculation showing that some dependency is still present [12]. 

In this context, G. Hernandez et al. investigated the microcirculation of a particular critically ill septic population: septic patients with arterial hypotension without elevated lactate concentrations. In an earlier study, these authors showed that persistent sepsis-induced hypotension without hyperlactatemia was associated with less severe organ dysfunction and a very low mortality risk (5.2 versus 17.4% for patients with lactate concentrations >2.5 mmol/L) [13]. In the present study, the authors used an SDF imaging device to study the microcirculation of 45 of these patients. There were relatively few abnormalities in this population, as shown by a median MFI value of 2.4 and a median percentage of perfused vessels of 87.3%. This study tends to support the notion that patients with persistent sepsis-induced hypotension without hyperlactatemia exhibit a distinctive clinical and physiological profile within the spectrum of septic shock. This subject should be addressed in future studies. 

A. P. C. Top et al. studied the behavior of the sublingual microcirculation after the start of ECMO therapy in neonates with severe respiratory failure. ECMO usually induces an improvement in hemodynamics and an immediate decrease in vasopressor needs. Nevertheless, beneficial cardiovascular effects after ECMO were not evident in this study as shown by unchanged blood pressure and no changes in infusions of vasoactive or inotropic drugs. Simultaneously, the sublingual microcirculation failed to improve and the alterations present at baseline remained present. In contrast, a group of patients on mechanical ventilation, with similar derangements at baseline, showed a decrease in microvascular perfusion over time. These findings suggest that ECMO could have a delayed effect on the microcirculation and thus prevent a further deterioration in microvascular flow. Unfortunately, the lack of cardiac output measurements precludes a fuller understanding of these results.

Finally, after works on measurements of the microcirculation at the bedside, studies on other compounds of the microcirculation, such as the glycocalyx or red blood cells (RBCs), are reported. Enzymatic degradation of the glycocalyx induces vascular leakage *ex vivo*, so S. A. Landsverk et al. investigated enzymatic treatment in an *in vivo* whole body hamster model. In addition to looking at the effects of degradation of the glycocalyx on endothelial leakage, these authors also investigated the potential effects of this process on the microcirculation. After injection of hyaluronidase, they measured plasma volume and functional capillary density as markers of the microcirculation. Enzyme treatment did not induce changes in plasma or albumin volumes, but reduced functional capillary density. There was no correlation between plasma hyaluronan concentrations and plasma volume or microcirculatory disturbances, despite a 50–100 fold increase in plasma hyaluronan. To explain their results, the authors suggest that impaired mechanotransduction associated with vasoconstriction, mainly due to loss of hyaluronan from the endothelial glycocalyx, was a possible mechanism [14]. Another possibility is the increased RBC rigidity at higher hyaluronan concentrations [15]. 

In another article, Y. Serroukh et al. comprehensively review the alterations in the erythrocyte membrane that occur in sepsis. This issue is potentially important to explain the microcirculatory abnormalities in sepsis. The authors discuss the alterations in the components of the RBC membrane that have previously been described. This membrane is essential for RBC deformability and rheology, and changes in the membrane and its complex interactions could significantly affect the microcirculation. Although clinical evidence is limited, RBC rheologic alterations in sepsis and their effects on blood flow and oxygen transport may have important implications, and improved understanding of the underlying mechanisms is important. Consequently, this review not only contributes to our understanding of current knowledge but also provides a framework for future research.

In conclusion, this special issue highlights the disturbances in the microcirculation in critically ill patients and presents some answers to important questions concerning methodology or particular populations of patients. These articles provide some additional pieces to the complex puzzle of optimizing treatment of the critically ill patient!

*Michael Piagnerelli* *Can Ince* *Arnaldo Dubin*
